# Metal Oxides Nanomaterials and Nanocomposite-Based Electrochemical Sensors for Healthcare Applications

**DOI:** 10.3390/bios13050542

**Published:** 2023-05-12

**Authors:** Palanisamy Kannan, Govindhan Maduraiveeran

**Affiliations:** 1 College of Biological, Chemical Sciences and Engineering, Jiaxing University, Jiaxing 314001, China; 2 Materials Electrochemistry Laboratory, Department of Chemistry, Faculty of Engineering and Technology, SRM Institute of Science and Technology, Kattankulathur 603 203, Tamil Nadu, India

**Keywords:** transition metal oxides, nanomaterials, nanocomposites, electrochemical sensors, healthcare monitoring, biomedical applications

## Abstract

Wide-ranging research efforts have been directed to prioritize scientific and technological inventions for healthcare monitoring. In recent years, the effective utilization of functional nanomaterials in various electroanalytical measurements realized a rapid, sensitive, and selective detection and monitoring of a wide range of biomarkers in body fluids. Owing to good biocompatibility, high organic capturing ability, strong electrocatalytic activity, and high robustness, transition metal oxide-derived nanocomposites have led to enhancements in sensing performances. The aim of the present review is to describe key advancements of transition metal oxide nanomaterials and nanocomposites-based electrochemical sensors, along with current challenges and prospects towards the development of a highly durable and reliable detection of biomarkers. Moreover, the preparation of nanomaterials, electrode fabrication, sensing mechanism, electrode-bio interface, and performance of metal oxides nanomaterials and nanocomposite-based sensor platforms will be described.

## 1. Introduction

Owing to their strong electrocatalytic activity, low price, high organic catching aptitude, reduced dimension, large surface-to-volume ratio, and high degree of crystalline nature, metal oxide nanocomposites have been extensively employed and are already established as an active electrocatalyst for sensing various emergent biomarkers [1,2,3]. Typically, the electrocatalytic properties of the metal oxide nanocomposites are closely related to the active sites, electrochemical active surface area, surface energy, etc. [4,5,6]. To attain high sensing performance properties of the metal oxides-based nanocomposites, the catalytic materials have been prepared as small as possible to grow more available active sites and available surface area [7,8,9]. 

However, because of their high surface energy, the ultra-small-sized nanoparticles are frequently less durable and effortlessly aggregate into larger particles, which may have an effect on the reduction of the specific surface area [10,11,12]. The most commonly employed method is the stabilization the nanocomposites by the use of capping agents, surfactants, polymers, and ligands. Still, the use of capping agents can effectively confine aggregation and harshly limit the catalytic activities [13,14]. Some other methods to producing defined compound oxides or other nanostructures such as nanoporous, nanotubular, nanorod, and nanowire morphologies are either template-based or template-free hydro- and solvo-thermal strategies [15,16,17].

Over the last decade, numerous metal oxide nanocomposites such as one-dimension (1D), two-dimension (2D), and three-dimension (3D) have exhibited outstanding catalytic and sensing properties [18]. In the field of catalysis and sensors, nano-dimensions have been employed aggressively because of their interesting performance in catalysis and structural stability when compared to bulk materials. The selection of preparation approaches for the production of dimension- and morphology-controlled preparation of transition metal oxides-based nanocomposites is a major prospect. The specific size, composition of materials, crystal lattices and structures, level of defects, electronic states, etc., of the metal oxides nanocomposites, is not so clear. It is a crucial challenge for understanding the connection between structural properties and electrocatalytic activities. The smart properties of metal oxides nanocomposites provide them with structural and crystalline flexibility for a wide range of electrochemical sensor applications. In addition, the exclusive characteristics of physicochemical and electrochemical properties of metal oxides nanocomposites may be easily altered by modifying the preparation settings. 

Previous research studies and reviews have reported that precious metal nanomaterials dispersed on numerous metal oxide supports as metal nanocomposites, which are catalytically more active for various electrochemical reactions [17,19,20,21,22]. In some cases, metal oxide nanomaterials are used as support materials for embedding noble metallic nanoparticles, and in particular Au, Pt, and Ru nanoparticles, dispersed on an oxide support often showed much higher catalytic activity than the single-component nanoparticles [10]. The enhanced catalytic activity is associated with the synergetic effect that occurs at the interface of metal and oxide support. It is supposed that the electronic structures can be altered via the deposition of noble metal on metal oxide nanomaterials as metal nanocomposites, offering growth to oxygen vacancies on the interfacial [4,13]. The solution dispensation of great excellence freestanding thin films via vacuum filtration, spin coating, drop casting, spray-coating, etc., shows a key role in various electrochemical sensor applications. The metal oxides nanocomposites film could demonstrate favourable environments as the catalytically active layers in sensor platforms. 

The recent investigations appear to indicate that small metal clusters in the heterostructures are the real active source for catalytic reactions. These tiny Au clusters possess low-coordinate Au atoms in comparison to bulk Au poly-crystal surfaces and take higher-energy d states, which are easily reactive and absorb/activate oxygen moieties [23]. In recent years, more robust and practical catalysts of metal oxide nanoparticles dispersed on graphene and carbon nanotubes-based electrochemical sensor systems with comparative or even better catalytic performance than noble metals [13,24,25,26,27,28,29]. Early transition metals supported on graphene as nanocomposites have fascinated great interest, which has a large volume of geometrical surface area, outstanding electrical conductivity, and decent chemical solidity [13,28,29]. 

The use of metal oxide nanomaterials and their nanocomposites has become increasingly predominant, owing to the inadequate approaches to upsurge the activity of an oxide containing a single metal [30,31]. The immobilization of enzymes onto the metal oxide/electrode surface is required because metal oxide nanocomposites offered a biocompatible environment for the enzymes to function on the electrode and improve the selectivity and sensitivity of the sensing electrode via enabling electron-transfer kinetics between the enzyme and the electrode. The resulting activity catalyses the electrochemical reduction or oxidation of the analyte [32,33,34]. In this review, key advancements, electrode fabrication, sensing mechanism, present challenges, and prospects of the transition metal oxide nanomaterials and nanocomposites-based electrochemical sensors towards the development of highly durable and reliable detection of biomarkers will be described. This review majorly comprised of three sections, including metal oxide nanomaterials, metal oxide nanocomposites with noble metals, polymers, Mxenes, and microelectrodes, and figure-of-merit of metal oxide nanocomposites for describing the advantages and account of various designs of the state-of-the-art electrochemical sensors with improved sensitivity and selectivity. 

## 2. Metal Oxide Nanostructures

In recent years, significant technological developments have been developed in the practice of pure metal oxides and multi-metal oxides nanomaterials in electroanalytical sciences due to their quantum confinement effect, their high surface area to volume ratios, biocompatibility, chemical constancy, surface action, and adaptable electron transport possessions [35,36,37]. Several research efforts were made into grasping rapid and reliable electrochemical assays and sensing systems for both laboratory and point-of-care applications. The applications of metal oxide nanomaterials have been directed to enhancements in sensitivity, discrimination activity, and the simultaneous ability for the analysis of proteins, nucleic acids, and small organic or biomolecules [38,39]. 

**Figure 1:**
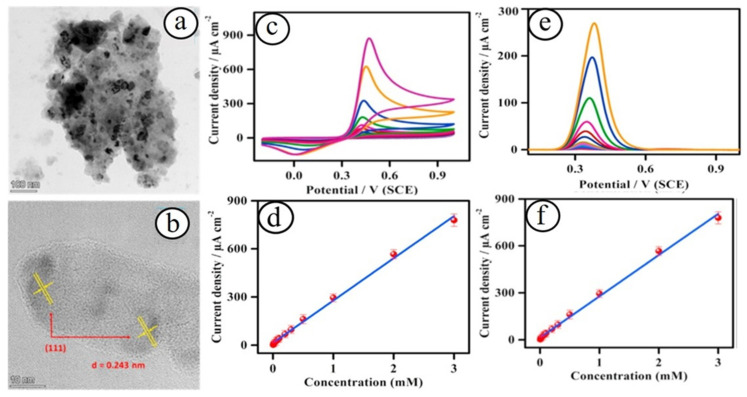
TEM (**a**) and HRTEM (**b**) images of NiO nanoparticles. Sensing study of 4-acetaminophen at the NiO-modified electrode through cyclic voltametric (**c**) and differential pulse voltametric (**e**) techniques with various concentrations of 4-acetaminophen, and their corresponding calibration plots (**d**,**f**) [40].

For instance, recently, Annandurai et al. developed a nickel oxide (NiO) nanoparticles-based electrochemical sensor platform for the detection of 4-acetaminophen in tablets and human blood serum samples [40]. Nickel oxide nanoparticles with an average particle size of 15–20 nm were synthesized using the hydrothermal method (Figure 1). The as-fabricated nickel oxide nanoparticles-based sensors exhibited a low detection limit of ~0.23 µM, high sensitivity of ~91.0 µA cm^−2^ mM^−1^, and a broad sensing range of 7.5 µM–3 mM. This electrochemical acetaminophen sensor also showed good selectivity in the presence of interfering species and good durability (up to 3000 s) and practical analysis of serum and tablet samples. Yang et al. reported a NiO nanomaterials-based enzyme-free electrochemical detection of glucose [41]. The prepared electrochemical sensor systems exhibited the highest glucose detection capability under an alkaline solution with a low detection limit of 0.32 μM (S/N = 3), high sensitivity of 2.037 mA mM^−1^ cm^−2^, a linear detection range from 4 μM to 7.5 mM, and good stability. Ahmad and co-workers recently developed an enzyme-free electrochemical uric acid sensor based on nano-berry morphological structured cobalt oxide (Co_3_O_4_) nanostructures with a low detection limit of ~2.4 µM (linear range of 5–3000 μM) and high sensitivity of 206 μA mM^−1^ cm^−2^ [42]. 

An enzyme-mimic hydrogen peroxide (H_2_O_2_) was developed by Morris et al. based on iron oxide (Fe_3_O_4_) nanodots modified with an indium tin oxide (ITO) electrode [43]. The resulting electrochemical sensor exhibited a low detection limit of 1.1 μM with a wide linear range (2.5 × 10^−3^–6.5 × 10^−3^). The sensor response reached a steady state signal within 5 s at the Fe_3_O_4_ nanodots/ITO electrode because of its large electrochemically active surface area and strong adsorption capability for immobilizing H_2_O_2_ on the electrode surface. The swift electron transfer among Fe^2+^ and Fe^3+^ ions created Fe_3_O_4_ nanodots as an exceptional electrical conductor. The as-developed Fe_3_O_4_ nanodots/ITO sensor showed good selectivity in the presence of various electrochemically active substances (glucose, uric acid, lactic acid, and ascorbic acid) and inorganic substances (NaCl, KCl, CaCl_2_, MgCl_2_, etc.) and stability (more than 12 days). 

Recently, Yan et al. demonstrated another interesting electrochemical cholesterol biosensor using thionine and Cu_2_O nanomaterials [44]. In this biosensor platform (cholesterol oxidase (ChOx)/thionine/cuprous oxide/glassy carbon electrode), nanostructured cuprous oxide (Cu_2_O) comprises an active pair of electron-hole with a large volume of active surface area, whereas thionine could enhance the efficacy of an electron transfer process among the enzyme and the electrode. The fabricated biosensor platform showed a LOD of ~1.8 nM with a wide sensing range (10–1000 µM) and high sensitivity of ~70.2 µA µM^−1^ cm^−2^ towards the detection of cholesterol. Numerous pure metal oxides nanomaterials derived from electrochemical sensors and biosensor platforms are reported for sensing and determining various biomarkers such as glucose, lactic acid, dopamine, ascorbic acid, etc. Owing to their biocompatibility, wider operating potential window, lower charging/background current, and electrochemical redox characteristics, mixed transition metal oxide nanomaterials are becoming more interesting and receiving more and more attention in electrochemical and bio-electrochemical sensing applications.

**Figure 2:**
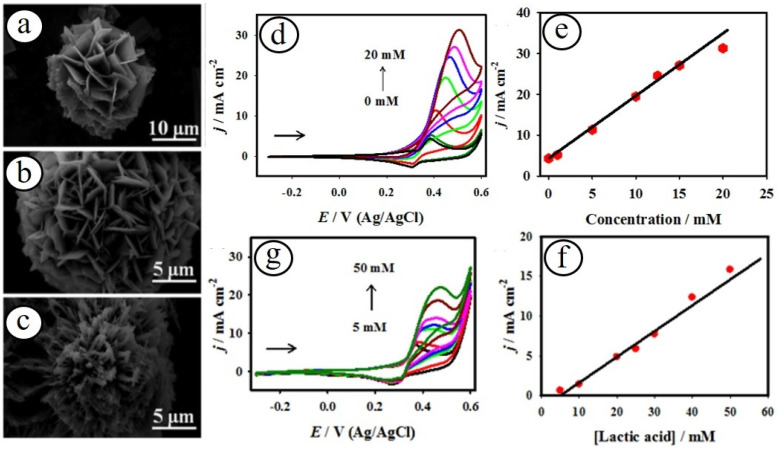
SEM images of the NiCo_2_O_4_ (**a**), NiO (**b**), and Co_3_O_4_ (**c**) nanomaterials. Sensing results for glucose (**d**) and lactic acid (**g**) with different concentrations, and their corresponding calibration plots (**e**,**f**), respectively [15].

In recent years, spinel-structured transition metal oxide nanomaterials have increased devotion in various catalytic and sensor applications, owing to their prominent catalytic activity, electrical conductivities, and crystalline stability [45,46,47]. In particular, cobaltite systems with a spinel crystalline structure (MCo_2_O_4_, where M = Mn, Ni, Fe, Cu, and Zn) exhibited interesting projections due to their outstanding catalytic and physicochemical properties [48]. A variety of nickel–cobalt oxide (NiCo_2_O_4_) nanostructured electrode materials have been developed with different morphologies such as nanoflowers, nanosheets, nanorods, nanowires, etc., towards the sensitive detection of various biomarkers [49,50,51,52]. Maduraiveeran and co-workers reported porous-structured three-dimensional (3D) NiCo_2_O_4_ nanoflowers as an enzyme-free electrocatalyst towards the direct oxidation of glucose and lactic acid under an alkaline electrolyte (Figure 2) [15]. 

The improved electrocatalytic activity of the NiCo_2_O_4_ nanostructures may be attributed to their exclusive morphological structure, small pore dimension, mixed active nickel and cobalt sites, huge electrochemically active surface area, and encouraging environment for the enhanced electron transfer kinetics. Recently, Karuppasamy and co-workers fabricated manganese cobaltite (MCO) nanoparticles based on non-enzymatic electrochemical detection of ascorbic acid in real pharmaceutical samples of Vitamin-C tablets and drinks [53]. The as-developed nano-enzymatic ascorbic acid sensor attained the low detection limit of 0.085 µM with a linear range of 5.0 μM–4.4 mM and good anti-interference activity.

Durai et al. reported mixed ternary NiCoZnO nanorods modified with glassy carbon electrodes for sensing dopamine in biofluids with a detection limit of 0.01 nM and a linear range from 1.0 nM to 0.5 µM [54]. The developed sensor successfully tested its sensing ability towards dopamine in human blood samples, showing good selectivity and high practical adaptability. The obtained sensing performance was due to its synergistic effect of NiCoZnO mixed metal oxides. In this sensor, NiO offered a huge amount of electrocatalytic active sites with multiple oxidation states (Ni^2+^/Ni^3+^), and ZnO and CoO provided exceptional chemical and mechanical stability. Other reliable electrochemical dopamine sensor-based mixed Al–Mn_0.65_–Cr_1.76_-oxide nanomaterials were developed by Alam et al. [55]. The fabricated Al–Mn_0.65_–Cr_1.76_-oxide nanomaterials sensor exhibited high sensitivity (~55.8 μA μM^−1^ cm^−2^), low detection limit (~96.8 pM), wide linear range (0.1 nM–0.01 mM), good selectivity, and long-term stability towards the detection of dopamine. Enzyme-free simultaneous sensing of acetylcholine and ascorbic acid using mixed ZnO/CuO nanoleaves oxides [56]. The simultaneous sensing of acetylcholine and ascorbic acid is highly required in the development of biological analysis, clinical diagnosis, and the field of healthcare. The developed mixed metal oxides of ZnO/CuO nanoleaves sensor demonstrated a low detection limit of (14.7 nM for acetylcholine and 12.0 pM for ascorbic acid) with a wide linear range of 100.0 pM–100.0 mM and high sensitivity (317.0 pAμM^−1^cm^−2^ for acetylcholine and 94.94 pAμM^−1^cm^−2^ for ascorbic acid). Various mixed metal oxides nanomaterials-based electrochemical biosensor platforms were demonstrated towards the detection of emerging biomarkers [57,58,59]. It is understood that one of the most exciting areas is the electroanalytical sensing of biomarkers with the support of transition metal oxide nanomaterials. The actual electronic conduction and rapid sensing response developed through the homogeneous distribution of active sites for attaining high sensing performance of the sensor. The morphology and dimension-controlled preparation of pure and mixed metal oxide nanomaterials are other interesting aspects for delivering improved sensitivity and selectivity, which can be achieved through various synthetic strategies. 

Most of the transition pure and mixed metal oxides nanomaterials may be employed as the sensor platforms for non-enzymatic sensing of glucose, lactic acid, hydrogen peroxide, dopamine, etc. (Table 1). Due to the low stability and high cost of enzymatic sensors, non-enzymatic electrochemical sensors attracted much attention. However, transition metal oxides nanomaterials-derived electrodes presented certain disadvantages, such as poor selectivity and low sensitivity. The mixed MCo_2_O_4_ spinel oxides and their composite nanomaterials have presented favourable sensing abilities, but there are still various routes to discover for practical sensor applications. For instance, two-dimensional MXenes, transition metal chalcogenides, phosphorous, etc., can be effectively employed to develop heterojunctions towards the detection of various biomarkers [60,61,62,63]. Transition metal oxide nanomaterials are, in principle, metal oxide materials in which a single unit is sized in the nanoscale domain, whereas transition metal oxide nanocomposites are engineered materials developed by combining them with noble metallic, carbon, polymeric, and biological nanostructures. By integrating several combinations of these nanostructures, a massive number of metal oxide nanocomposites may be established with outstanding catalytic and sensing characteristics due to their various features such as size, shape or morphology, conducting support, coordination site, composition, electron confinement, and distance between the inter-particles (Table 2). 

## 3. Metal Oxide Nanocomposites

### 3.1. Noble Metal and Metal Oxide Nanocomposites

**Figure 3:**
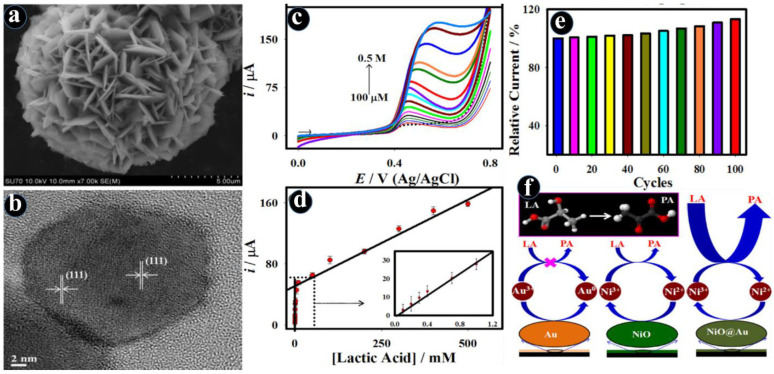
SEM (**a**) and HRTEM (**b**) images of the flower-like NiO nanostructures; electrochemical detection of lactic acid with different concentrations using DPV method (**c**); corresponding calibration plot (**d**) (Inset: Expanded view); durability of the NiO@Au electrode towards the lactic acid oxidation with various cycles (**e**); and electrochemical lactic acid sensing mechanism on the electrode (**f**) [68].

Owing to synergistic interactions, multiple active sites, and intrinsic catalytic activity, the nanocomposites of transition metal oxides with metal (Au, Pt, Ag, and Pd) nanoparticles, polymeric materials, and carbon (carbon nanotubes (CNTs), graphene, and fullerene) derived nanomaterials offer amplified electrochemical properties in comparison to the original constituents [64,65,66,67]. Accordingly, the electrochemical sensors not only prompt the detection limits with sub-femto molar but also prosper in an extensive sensing range and good selectivity for the onsite analysis of biomarkers. Maduraiveeran et al. developed the high-performance electrochemical lactic acid sensor based on enzyme-mimic gold-modified flower-like nickel oxide (NiO@Au) nanocomposites [68]. The Au deposition on NiO nanostructures can enhance electron transport properties because of its strong electronic and catalytic characteristics. This study revealed that Au was employed as a signal-improving element, while flower-like NiO nanostructures mimicked enzyme activity towards the lactic acid oxidation and functioned as an exceptional backing to enable Au deposition. The homogeneous distribution of Au on flower-like NiO nanostructures improved electron-transfer kinetics and exhibited a low limit of detection (11.6 μM), high sensitivity (8.0 μA/mM), broad linear range (100.0 μM–0.5 M), and good selectivity towards numerous interfering biomolecules, including cystamine, ascorbic acid, uric acid, and glucose (Figure 3) [68]. Furthermore, the resulting sensor was tested in human practical serum and urine samples for the detection of lactic acid.

It has been reported that Au-dispersed NiCo_2_O_4_ exhibited a low detection limit (5.8 μM) and high sensitivity (44.86 µA µM^−1^ cm^−2^)) towards sensing glucose compared to Ag-dispersed NiCo_2_O_4_ nanostructures [40]. According to density functional theory (DFT) analysis, which revealed that the Au nanoparticle was more strongly bound to NiCo_2_O_4_ than Ag nanoparticles, Au–NiCo_2_O_4_ led more conductively than Ag–NiCo_2_O_4_. Besides the sensing glucose, a variety of electrochemical sensors based on metal/metal oxide nanocomposites and binary metal oxide/metal oxide nanocomposites have been demonstrated for the detection of various biomarkers, including hydrogen peroxide, lactic acid, dopamine, NADH, ascorbic acid, paracetamol, cysteine, etc. [69,70,71]. The coherent design of hierarchical nanostructures, also called core@shell, has been employed for improving the electrochemical sensing behaviour, which will offer good electrode kinetics with large ECSA and abundantly accessible sites. 

### 3.2. Polymer and Metal Oxide Nanocomposites

Recent studies showed that PANI-derived electrochemical sensor practices have certain characteristic demerits including poor selectivity and reproducibility. PANI is often integrated with metal nanoparticles and metal oxide nanomaterials to overcome those challenges. Over the past decade, numerous transition metal oxide nanocomposites with polymeric materials have fascinated the field of electro-analysis [65,66,72]. Ponnaiah et al. developed sensitive electrochemical uric acid sensors based on polyaniline (PANI) coupled with silver-doped iron oxide (Ag–Fe_2_O_3_) nanostructures [73]. The as-prepared PANI-Ag–Fe_2_O_3_ nanocomposites electrode delivered a low detection limit of 102 pM and a wide linear range of 0.001–0.900 μM. In addition to PANI polymeric materials, polypyrrole (PPy) and poly(3,4- ethylenedioxythiophene) (PEDOT) were successfully integrated with metal oxide nanomaterials for sensing biomarkers for improving their electrical conductivity [71,74,75]. 

Owing to a wide operating potential window, strong surface chemistry, lower price, and chemical inertness, various carbon-derived nanocomposites of metal oxides with graphite, carbon paste (CP), carbon nanotubes (CNTs), graphene (GR), carbon fibers (CFs), activate porous carbon, and carbon nanodots are usually utilized as the sensing materials for the sensitive detection of a variety of biomarkers [14,27,76,77,78,79]. Recently, Wahab et al. reported a peroxidase mimicking electrochemical glucose sensor based on nanoarchitectured iron oxide embedded in mesoporous carbon nanozymes [80]. The resulting nanocomposites electrode exhibited enhanced homogeneous distribution of iron active sites on 3D mesoporous carbon support with a large volume of electrochemically active surface area, which may lead to improved analyte adsorption and high electron mobility and prominently improved peroxidase mimetic activity. This sensor exhibited a low LOD of 2 µM in the spiked sample.

**Figure 4:**
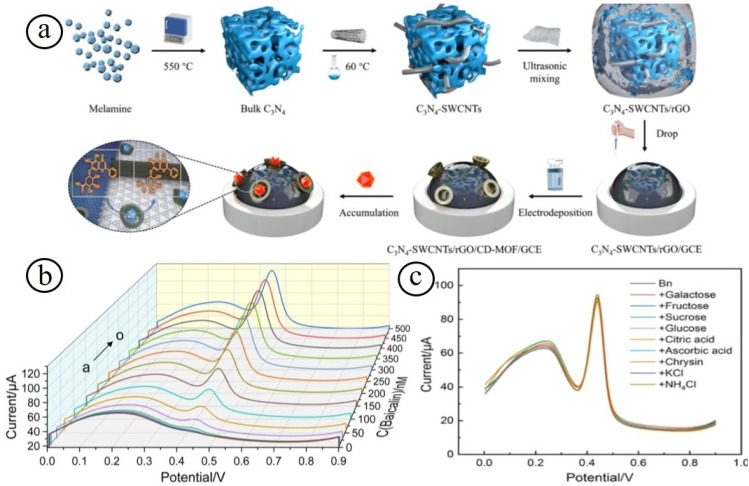
Schematic illustration of the fabrication of nanocomposites electrodes (**a**); the sensing response with different concentrations (**b**), and selectivity (**c**) of the nanocomposites towards the detection of baicalin [81].

Another interesting electrochemical baicalin (Bn) sensor was established by Zhao et al. based on graphitized carbon-nitride single-walled carbon nanotube nanocomposites (C_3_N_4_-SWCNTs), reduced graphene oxide (rGO), and an electrodeposited cyclodextrin-metal organic framework (CD-MOF) [81]. The resulting sensor based on C_3_N_4_-SWCNTs/rGO/CD-MOF nanocomposites attained the sensitive and selective detection of Bn with a broad linear range of 1.0 nM–0.5 µM and low LOD of 0.46 nM with a sensitivity of 220 A/M (Figure 4). In addition, the sensor demonstrated satisfactory durability and reproducibility for sensing Bn in real human serum and bear bile Scutellaria eye drops samples. Carbon nanomaterials-based metal oxide nanocomposites have fascinated the area of electrochemical sensors because of their high robust sensing performance [82,83,84]. The coherent strategy of hierarchical nanostructured metal oxide nanocomposites is employed to enhance the electrochemical sensing performance in terms of catalytic activity, rapid response, low detection limit, high sensitivity, good selectivity, and practicability, since the nanocomposites may offer a good electron transfer kinetics, high quantity of electrochemical active sites, mixed oxidation state, and coordination environment. 

### 3.3. MXenes and Metal Oxide Nanocomposites

Two-dimensional (2D) transition metal (TM) carbides, nitrides, and carbonitride, also termed as MXenes, have been considered to be promising materials with the integration of metal oxides nanocomposites for constructing sensors systems [85]. In general, metal oxides nanomaterials that stayed on MXene nanostructures as a nanocomposite via van der Waals interactions are highly well-organized and have self-assembling characteristics that may effortlessly control their packing or thin film. The nanocomposites of metal oxides-MXene materials often show the integrated merits of both materials of metal oxides and MXene. In several cases, MXene 2D-sheets aid as the conductive materials to build or engineer the metal oxides nanomaterials, often facilitating fast electron and ion transfer kinetics to avert the aggregation and improve the interfacial active sites of metal oxides nanomaterials in various electrochemical applications [86]. 

Park and co-workers demonstrated an electrochemical biosensor platform based on nickel oxide (NiO)–reduced graphene oxide (rGO)/MXene nanocomposites for sensing active influenza viruses (H1N1 and H5N2) and viral proteins [87]. The detection principle is based on the signal inhibition, i.e., the specific interaction between H1N1 (QMGFMTSPKHSV) and H5N1 (GHPHYNNPSLQL) binding peptides, which bound on the NiO–rGO/MXene electrode materials. The existence of viral surface protein hemagglutinin (HA) may be a key role in reducing the signal of the sensor. In this method, the as-developed nanocomposites of NiO–rGO/MXene can be integrated with effects in the signal, such as low electrochemical resistance, porous nature, and electrochemical active sites, thereby facilitating the adsorption of viruses. The resulting electrochemical biosensor based on the NiO–rGO/MXene nanocomposites demonstrated a low detection limit of 3.63 nM for H1N1 and 2.39 nM for H5N1, respectively, and good specificity. The performance of the developed biosensors has possessed the capability of ultra-sensitive and selective detection of influenza viruses and viral proteins, evolving a lot of choices of clinical screening methods for sensing affected patients.

In addition, metal oxides dispersed on conducting polymers-based nanocomposites have been employed as the sensing electrode materials for biomarkers [88,89]. A variety of preparation strategies and electrode preparation methods have been reported due to their vast field of applications in electrocatalysis, separation devices, sensors, etc. In particular, the conducting polymers include polyaniline (PANI), polyacetylene (PA), polypyrrole (PPY), polythiophene (PTH), and poly(3,4-ethylenedioxythiophene) (PEDOT) and their metal nanocomposites were effectively used for electrochemical sensing biomolecules. For instance, Fayemi et al. reported polyaniline/NiO, ZnO, and Fe_3_O_4_ nanocomposites-based electrochemical sensor platforms for detecting dopamine in the presence of ascorbic acid (AA) and serotonin (SE) under physiological pH 7.0 [90]. The as-developed PANI–metal oxides (MO) nanocomposites sensor exhibited a fast electron transfer process and improved the electrochemical response for the oxidation of dopamine. The sensor exhibited the limit of detection of 0.0153 μM with a sensing range of 2.4–20.0 μΜ. The resulting sensor showed good selectivity in the presence of electrochemically active species of ascorbic acid and serotonin. It is found that there are synergies between metal oxides and electrical conducting polymers of PANI. In recent years, various photoelectrochemical (PEC) sensors have been developed using metal oxide nanocomposites for various in vitro diagnostics [91,92,93]. 

**Figure 5:**
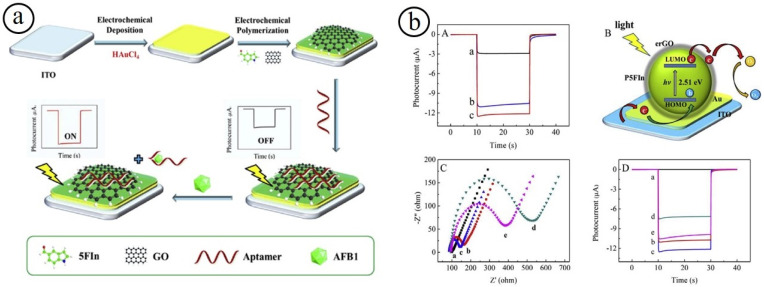
(**a**) Pictorial representation of the PEC aptasensor; sensing response of PEC of the various electrode materials under 0.1 M PBS (pH 7.4). (**b**) The sensing response with various concentrations (**A**); generation of photocurrent mechanism erGO/P5FIn/Au modified electrode (**B**); EIS response with various concentrations (**C**) and time-based photocurrent response curves of the modified ITO electrodes with various concentrations (**D**) [91].

The design of a PEC sensor is also considered to be a new detection approach, which has attracted more attention [92,93]. In the PEC strategy, UV- or visible-light-radiation plays as the excitation source and the developed photocurrent is employed as a sensing signal. Owing to the combined advantages of photoirradiation and electrochemical signal output, the PEC-based detection method often exhibits higher sensitivity with low charging current, playing a crucial role in various chemical and biological analyses. The systematic design of carbon-based nanocomposites consisting of metal oxides, conducting polymers, and noble metal particles is an interesting strategy for the establishment of biosensor platforms. For instance, Zhang et al. developed a simple signal-on PEC aptasensor for the detection of aflatoxin B1 (AFB1) using electrochemically reduced graphene oxide/poly(5-formylindole)/AuO (erGO/P5FIn/Au) nanocomposites (Figure 5) [91,92]. The prepared nanocomposites exhibited an excellent sensing performance towards the detection of AFB1. In the PEC aptasensor, the conducting polymers of Poly(5-formylindole) (P5FIn) produce electron-hole pairs using irradiation of light, which usually leads to forming a robust cathode photocurrent. The utilization of noble metal/metal oxides may be played as the PEC sensor amplifier and the erGO employed to embed AFB1 aptamer chain through π–π stacking interaction among a carbon six-membered ring in graphene and the C-N heterocyclic ring in nucleobases of ssDNA. The resulting PEC sensor demonstrated a low detection limit of 0.02 pg mL^−1^, a broad sensing range (0.01–100 ng mL^−1^), good specificity, and practicability. The effectual design of PEC sensing materials with high sensitivity and selectivity, non-toxic, low-energy excitation, and low expense is a central challenge.

### 3.4. Metal Oxide Nanocomposites-Based Microelectrodes

**Figure 6:**
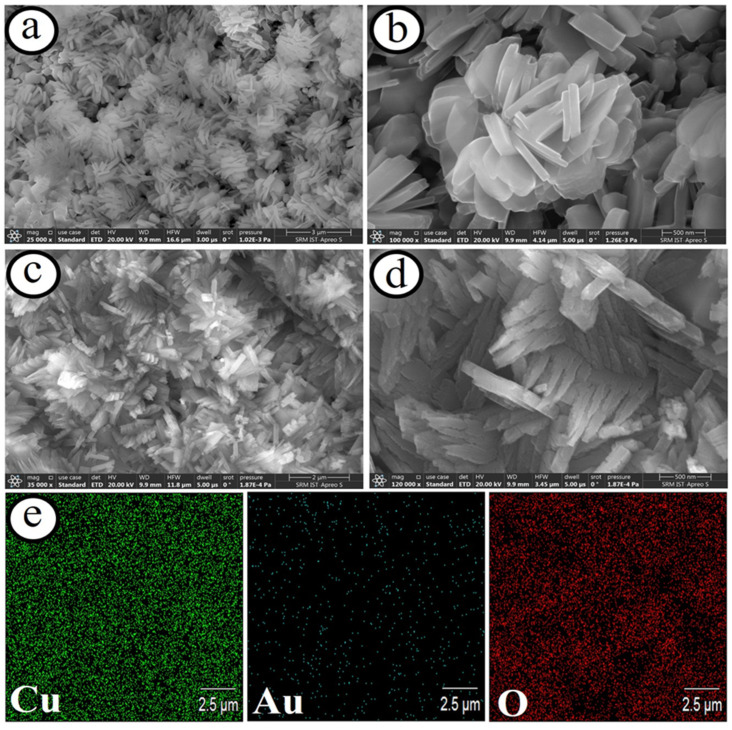
SEM images of the CuO microflowers on copper microelectrode (**a**,**b**), and Au dispersed CuO microflowers on microelectrodes (**c**,**d**) at low and high magnifications. Elemental mapping results of copper, gold, and oxygen elements of the microelectrodes (**e**) [94].

**Figure 7:**
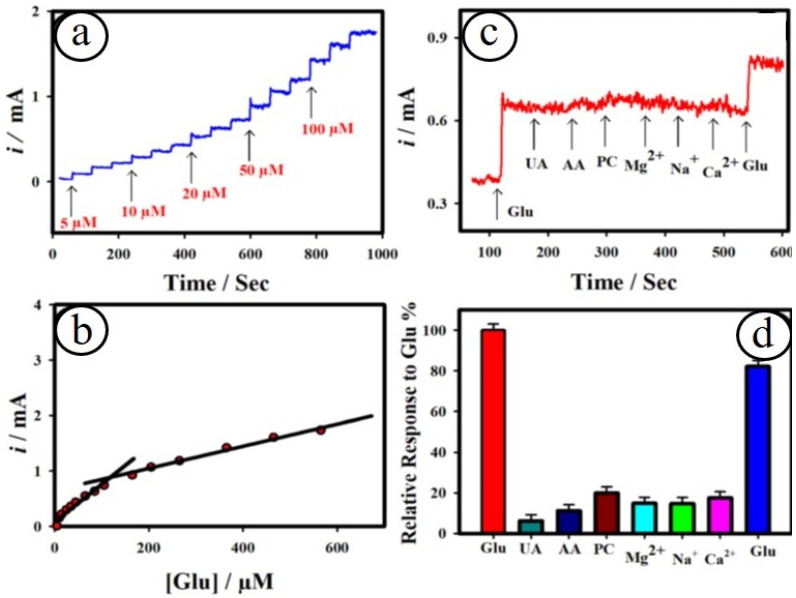
Amperometric sensing of glucose at the nanocomposites of Au@CuO MFs on copper microelectrodes (**a**); corresponding calibration plot (**b**); selectivity study at the Au@CuO MFs on copper microelectrodes at the applied potential of ~0.6 V (**c**). The relative response plot towards the detection of glucose in the existence of interferences (**d**) [94].

The design of miniaturized electrochemical biosensor systems for the detection of promising biomolecules in human body fluids becomes crucial, owing to their merits of high sensitivity and selectivity. The methods for the development of microelectrodes are one of the smart approaches for enhancing the signal-to-noise ratio via providing high faradaic current and small IR drop. A lot of microelectrodes based on carbon (C), gold (Au), platinum (Pt), boron-doped diamond (BDD), etc., have been established for detecting biomolecules [94,95,96]. For instance, Maduraiveeran and co-workers recently reported a self-supported and uniform dispersion of gold on the copper oxide microflowers on copper microelectrodes (Au@CuO MFs|CME) for the detection of both glucose and lactic acid in human serum and urine samples [94]. This novel strategy for the preparation of nanocomposites of Au@CuO MFs|CME involves the direct growth of flower-like passivated copper microelectrodes in nitric acid followed by the galvanic replacement of copper atoms with gold atoms (Figure 6). This method avoids the presence of surfactant or polymer without the use of any catalysts or complicated procedures for the preparation of Au@CuO MFs|CME nanocomposites. The resulting microsensor platform showed a broad detection range (5.0 μM–0.5 mM) with a LOD of ~1.41 μM and a sensitivity of ~4.14 mA μM^−1^ cm^−2^ for glucose and a linear range (100 nM–88.0 μM) with a LOD of ~27.0 nM and a sensitivity of ~6.19 mA μM^−1^ cm^−2^ (Figure 7). The as-developed microsensor based on Au@CuO MFs|CME nanocomposites delivered low sensing limits, high sensitivity, good specificity, and huge practical applicability for sensing both glucose and lactic acid in human serum and urine samples. Moreover, it is found that the specific and simultaneous sensing of glucose or lactic acid is a key challenge. Table 2 summarized the transition metal oxides nanocomposites materials-based electrochemical sensor platforms for sensing numerous biomarkers. 

**Table 2:** List of the electrochemical sensors reported based on metal oxide nanocomposites for sensing various biomarkers.

S. No	Electrode Material	Analyte	Real Sample	LOD	Sensitivity	Linear Range	Ref.
1.	NiO@Au	Lactic acid	Human serum and Urine	11.6 μM	8.0 μA mM^−1^	100.0 μM–0.5 M	[64]
2.	Au- NiCo_2_O_4_	Glucose	-	5.8 μM	44.86 µA µM^−1^ cm^−2^	5.8 μM–0.1 mM	[36]
3.	Ag–Fe_2_O_3_/PANI	Uric acid	Human blood and Urine	102 pM	-	0.001–0.900 μM	[69]
4.	Fe_2_O_3_/Meso Carbon	Glucose	-	2 µM	-	25 μM–10 mM	[76]
5.	C_3_N_4_-SWCNTs/rGO/CD-MOF	Baicalin	Human serum and Eye drops	0.46 nM	220 A M^−1^	1.0 nM–0.5 µM	[77]
6.	NiO–rGO/MXene	Influenza viruses H1N1 H5N1	Human plasma	3.63 nM 2.39 nM	- -	- -	[83]
7.	PANI/NiO	Dopamine	Injection	0.0153 μM	-	2.4 μM–20.0 μΜ	[86]
8.	erGO/P5FIn/AuO	AFB1	Peanuts and wheat samples	0.02 pg mL^−1^	-	0.01–100 ng mL^−1^	
9.	Au@CuO MFs|CME	Gucose Lactic acid	Human serum and Urine	1.41 μM 27.0 nM	4.14 mA μM^−1^ cm^−2^ 6.19 mA μM^−1^ cm^−2^	5.0 μM–0.5 mM 100 nM–88.0 μM	[90]
10	ZnO/MXene/GOx	Glucose	Sweat	17.0 μM	29 μA mM^−1^ cm^−2^	0.05–0.7 mM	[97]
11	GOx/Hemin@NC-ZIF	Glucose	-	10.0 μM	-	1.0–24 mM	[98]

SWCNTs: single-walled carbon nanotube; rGO: reduced graphene oxide; CD: cyclodextrin; P5Fin: poly(5-formylindole); erGO: electrochemically reduced graphene oxide; AFB1: aflatoxin B1; MFs: microflowers; CME: copper microelectrode; GOx: glucose oxidase; NC: nanocage; ZIF: zeolite imidazole.

## 4. Figure-of-Merit of Metal Oxide Nanocomposites

The design and establishment of high-sensing performance of durable and less-expensive sensing elements are of enormous importance for the growth and practical applications of electrochemical sensors and biosensor systems. Over the recent years, a lot of advanced and functional nanocomposites of metal oxides materials have been reported with high sensitivity, good specificity and reproducibility, and high practicability (Table 1 and Table 2). The electrocatalytic effect and selective sensing of emergent biomarkers with low-cost sensor platforms often show key roles in the advancement of sensor technologies. In the current scenario, the metal oxides nanocomposites consisting of high dispersion of third-row noble transition metals (gold, platinum, palladium, silver, etc.), carbon nanoallotropes (carbon nanotubes, graphene, graphene oxide, reduced graphene oxide, fullerene, carbon dots, carbon fibers, etc.), and polymer materials (conducting polymers and biopolymers) derived electrode materials are highly prime for the construction of electrochemical sensor and biosensor platforms for clinical and biomedical applications. Although, the practical industrial prospects are harshly hindered because of their low abundance and high cost of the state-of-the-art gold and platinum-based sensing elements. In this regard, the design of enormously catalytically active, prompt sensing performance and high biocompatible metal oxides nanocomposites-derived candidates has become emergent in electrochemical sensors systems. 

In recent years, metal oxides nanocomposites have been attractive in various electrochemical technologies, in particular, in biosensors and bioelectronics. Especially, due to the characteristic electronic structure, high catalytic sites, and maximum atom utilization efficiency, single-atom catalysts (SAC) based on iron, nickel, and copper oxides are considered to be promising sensing elements. The high utilization of metal atoms in the electrode materials makes them deliver an outstanding sensing performance with lower consumption and a reduction of the cost of the electrode materials. In general, SACs are often dispersion on carbon nanomaterials or polymeric nanomaterials. The high sensing performance of the detection elements based on metal oxides nanocomposites is mainly attributed to various features such as size, shape or morphology, conducting support, coordination site, composition, electron confinement, and distance between the inter-particles. 

## 5. Conclusions and Outlook

Transition metal oxide-derived nanomaterials and their nanocomposites provide an excessive potential for the progress of high-performance electrochemical sensors and biosensor platforms. Owing to improved detection performance with rapid response, low detection limit, high sensitivity, and potential selectivity in the presence of a real complex environment and robust analysis, metal oxides and their nanocomposites established widespread consideration. The excellent characteristics of metal oxides-based nanocomposites make them attractive as sensing elements with high surface area to volume ratios, biocompatibility, chemical stability, surface reaction activity, and adjustable electron transport properties. The unique electronic and catalytic properties of metal oxide-based nanocomposites are therefore able to increase the sensitivity, selectivity, and stability of the sensor platforms towards the detection of biomarkers. The present review offers major advancements in transition metal oxide nanomaterials and their nanocomposites-based electrochemical sensor and biosensor platforms for healthcare monitoring. Subsequently, the facile sensing electrode fabrication, catalytic activity, reaction mechanism, hetero-structured active sites, and surface engineering for enhancing transducer performance are described. 

Transition metal oxides-based nanocomposites with numerous 1D- or 2D- or 3D-nanostructures, including nanowires, nanotubes, nanoribbons, nanosheets, and nanodots are enduring to be at the lead of nanoscience and nanotechnology for electrocatalytic and sensor applications due to their unique physicochemical and electrochemical characteristics, which are associated to their dimensional anisotropy. The versatile utilization of nanostructured metal oxides and their nanocomposites in efficient electrocatalytic sensor applications has established worldwide concern and amplified investigative attention in commercial analytical applications towards clinical and biomedical diagnostics and healthcare monitoring. The enhancement of their overall sensing recital, primarily including the activity, selectivity, and solidity, is suggestively contingent on the progress of new composite materials employed as sensing elements. It is commonly understood that surface/interface reactions are crucial in several electrochemical processes, from electrochemical processes on an electrode to heterogeneous catalytic reactions. Hence, researchers prefer to synthesize smaller size and hetero-structured nanocomposites materials to ensure that larger active surfaces and multiple active sites are exposed in the selective electrocatalytic reactions. 

In-depth explorations are required to address challenges, including stabilization of multiple active sites, optimizing the adsorption of analytes and adsorbed product intermediates, reusable sensors platforms, etc. In recent years, heterostructures or nanocomposites of catalytically active sites dispersed on a durable matrix without allowing for agglomeration have emerged and appeared as excellent candidates to attain sensitive and selective detection of biomarkers via stabilization and tuning of electrochemically active sites. Finally, it is believed that further investigations may be studied for correlating the relationship between morphological/crystalline structures of the metal oxides nanocomposites and the reaction mechanism of analytes. Consequently, an organized inspection of the effect of morphological and crystalline structures, coordination environment, synergistic effects of multiple active elements, double layer interface, etc., of the transition metal oxides and their nanocomposites on the electrocatalytic and sensing performance towards clinical, biomedical, healthcare monitoring, and biological applications desires examination.

## Figures and Tables

**Table 1:** List of the electrochemical sensors reported based on pure metal oxide and mixed metal oxides nano materials for detecting biomolecules.

S. No.	Electrode Material	Analyte	Real Sample	LOD	Sensitivity	Linear Range	Ref.
1.	NiO NPs	4-Acetaminophen	Tablet and Serum	0.23 µM	91.0 µA cm^−2^ mM^−1^	7.5 µM–3 mM	[36]
2.	NiO NMs	Glucose	-	0.32 μM	2.037 mA mM^−1^ cm^−2^	4.0 μM to 7.5 mM	[37]
3.	Co_3_O_4_ NBs	Uric acid	Urine	2.4 µM	206 μA mM^−1^ cm^−2^	5.0–3.0 mM	[38]
4.	Fe_3_O_4_ NDs	H_2_O_2_	Serum	1.1 μM	-	2.5–6.5 mM	[39]
5.	Cu_2_O/ChOx/TH	Cholesterol	-	1.8 nM	70.2 µA µM^−1^ cm^−2^	10.0– 1000 µM	[40]
6.	NiCo_2_O_4_ Nanoflowers	Glucose Lactic acid	- -	1.0 mM 5.0 mM	2.6 mA mM^−1^ cm^−2^ 0.43 mA mM^−1^ cm^−2^	1.0–20.0 mM 5.0–50.0 mM	[13]
7.	MCO NPs	Ascorbic acid	Tablets and Drinks	0.085 µM	-	5.0 μM–4.4 mM	[49]
8.	NiCoZnO Nanorods	Dopamine	Blood	0.01 nM	-	1.0 nM–0.5 µM	[50]
9.	Al–Mn_0.65_–Cr_1.76_-oxide	Dopamine	-	96.8 pM	55.8 μA μM^−1^ cm^−2^	0.1 nM–0.01 mM	[51]
10.	ZnO/CuO Nanoleaves	Acetylcholine Ascorbic acid	- -	14.7 pM 12.0 pM	317.0 pA μM^−1^ cm^−2^ 94.94 pA μM^−1^ cm^−2^	100 pM–100 mM 100 pM–100 mM	[52]

NPs: nanoparticles; NMs: nanomaterials; NBs: nano-berry; NDs: nanodots; ChOx: cholesterol oxidase; TH: Thionine; MCO: manganese cobaltite.

## Data Availability

Not applicable.
